# Rapid eye movement sleep and slow wave sleep rebounded and related factors during positive airway pressure therapy

**DOI:** 10.1038/s41598-021-87149-3

**Published:** 2021-04-07

**Authors:** Jin-Xiang Cheng, Jiafeng Ren, Jian Qiu, Yingcong Jiang, Xianchao Zhao, Shuyu Sun, Changjun Su

**Affiliations:** Department of Neurology, Tangdu Hospital, Fourth Military Medical University, Xi’an, 710038 Shaanxi Province China

**Keywords:** Medical research, Neurology

## Abstract

This study aimed to investigate the clinical characteristics and predictors of increased rapid eye movement (REM) sleep or slow wave sleep (SWS) in patients with obstructive sleep apnea (OSA) following positive airway pressure (PAP) therapy. The study retrospectively analyzed data from patients with OSA who underwent both diagnostic polysomnography (PSG) and pressure titration PSG at the Tangdu Hospital Sleep Medicine Center from 2011–2016. Paired diagnostic PSG and pressure titration studies from 501 patients were included. REM rebound was predicted by a higher oxygen desaturation index, lower REM proportion, higher arousal index, lower mean pulse oxygen saturation (SpO_2_), higher Epworth sleepiness score and younger age (adjusted R^2^ = 0.482). The SWS rebound was predicted by a longer total duration of apneas and hypopneas, lower N3 duration, lower SpO_2_ nadir, lower REM proportion in diagnostic PSG and younger age (adjusted R^2^ = 0.286). Patients without REM rebound or SWS rebound had a high probability of comorbidities with insomnia and mood complaints. Some parameters (subjective and objective insomnia, excessive daytime sleepiness, age and OSA severity) indicate changes in REM sleep and SWS between diagnostic and titration PSG tests. Treatment of insomnia and mood disorders in patients with OSA may helpful to improve the use PAP.

## Introduction

Obstructive sleep apnea (OSA) is characterized by sleep fragmentation due to recurring episodes of upper airway obstruction with frequent oxygen desaturation. Cortical arousal is often necessary to reopen the obstructed airway and restore breathing. The sleep architecture of OSA is characterized by increased N1 and N2 sleep stages and reduced slow wave sleep (SWS) and rapid eye movement (REM) sleep^1^.

Positive airway pressure (PAP) therapy is the first recommended treatment for adults with OSA^2^. The main principle of PAP treatment is mainly positive pressure ventilation to open the airway obstruction during sleep, thereby reducing hypoxia caused by airway obstruction. In its earliest use PAP treatment was found to not only reduce respiratory events and increase blood oxygen saturation but also to reduce non-rapid eye movement (NREM) sleep stage 1 and 2 and increase SWS and REM sleep^3^. A significant increase in SWS and REM sleep, known as rebound, is commonly observed after sleep fragmentation and sleep deprivation^3–5^. With the popularity of PAP as an OSA treatment, some patients with OSA have experienced reduced respiratory events and show restored sleep architecture during PAP treatment. Many patients who were treated with PAP had difficulty falling asleep and maintaining sleep, with decreased SWS and REM sleep^6–11^. The initial use of PAP equipment is similar to a polysomnography (PSG) examination, which may lead to the first night effect, resulting in worse sleep quality, difficulty falling asleep, increased wake after sleep onset (WASO) and decreased sleep efficiency (SE)^8^. Additionally, insomnia, anxiety, and depression are common comorbidities of OSA^12–15^ that may lead to changes in sleep architecture, such as a long sleep latency, long WASO, increased N1 and N2 sleep stages, decreased SWS, and decreased or variable REM sleep^16,17^. Increased N1 and N2 sleep stages, decreased SWS, and decreased or variable REM sleep are common changes observed in patients with OSA, insomnia, anxiety, and depression^16,17^. PAP therapy only reduces respiratory events by providing positive airway pressure, thus reducing the awakening caused by respiratory events and promoting the recovery of sleep architecture, but has no immediate therapeutic effect on insomnia, anxiety, and depression. Because the first time use of PAP equipment aggravates insomnia and anxiety in patients, leading to decreased sleep quality, PAP treatment in patients with OSA fails. Which factors will affect the recovery of REM sleep and SWS sleep in patients with OSA who are treated with PAP? Few previous studies have established the rebound model of REM sleep and SWS in patients with OSA receiving PAP treatment, but clear statistical criteria for the definition of REM and SWS rebound and a comparison of clinical differences between REM and SWS rebound classifications are lacking^8–11^.

The first objective of this study was to objectively define REM and SWS rebound. The second objective was to determine the prevalence of REM and SWS rebound during PAP titrations and the clinical characteristics of REM sleep and SWS with or without rebound. The third objective was to establish a model for predicting changes in the REM sleep and SWS.

## Methods

### Patients

**Figure 1:**
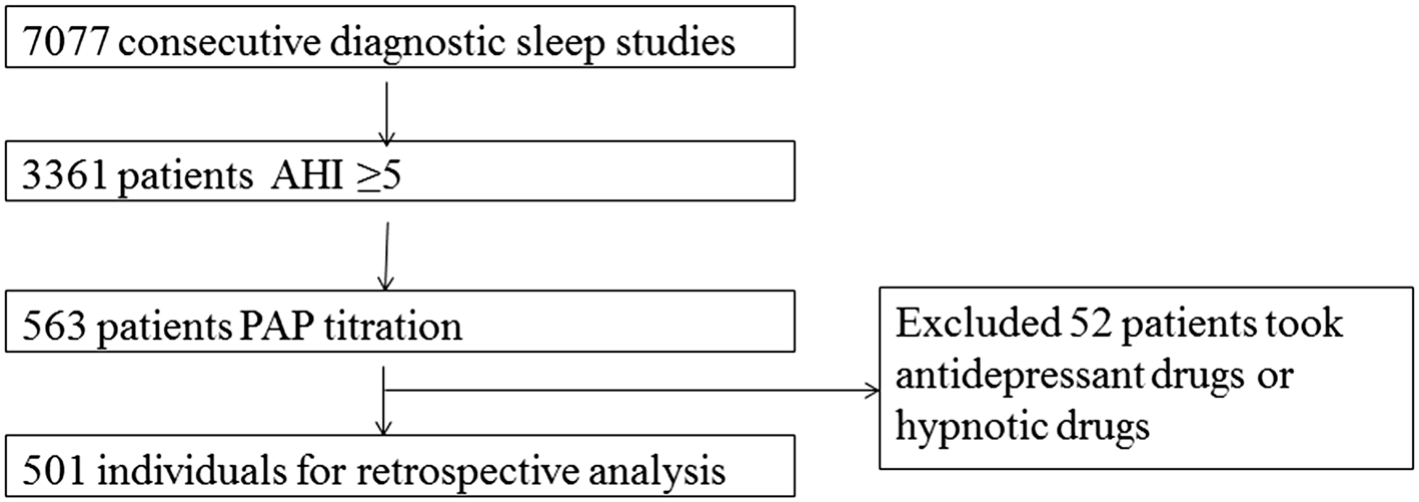
Flowchart for study selection.

Between January 2011 and December 2016, 7077 consecutive diagnostic sleep studies were performed at the Tangdu Hospital Sleep Laboratory, and the apnea–hypopnea index (AHI) was ≥ 5 in 3361 studies. PAP titration was ordered for 563 patients. Patients using sleep drugs and antidepressants within half a month of undergoing sleep studies or PAP studies were excluded, and thus data from 501 individuals were available for this retrospective analysis (Fig. 1). All procedures involving patients were performed in accordance with the ethical standards of the institutional and/or national research committee and the 1964 Declaration of Helsinki and its later amendments or comparable ethical standards. The study was approved by the Independent Ethics Committee of the Institution for National Drug Clinical Trials, Tangdu Hospital, Fourth Military Medical University (approval number: 201912-41). As this study employed a retrospective design with no violation of patient privacy, the ethics committee waived the requirement for informed consent.

### Procedure

The following patient information was collected: age, sex, body mass index (BMI), Epworth Sleepiness Scale (ESS), clinical symptoms, and comorbidities. Clinical symptoms and comorbidities such as insomnia, anxiety, and depression were determined by self-report.

### Polysomnography and pressure titration

All the patients spent two nights in a sleep laboratory: one night for diagnostic PSG, and the other for PSG and PAP titration. Diagnostic PSG was performed using a computerized PSG system (Alice 4 or 5; Respironics, Pittsburgh, PA, USA). The recording montage included an electroencephalogram, electrooculogram, electromyogram, electrocardiogram, breathing effort, airflow, oximetry, and body position. The starting mode was continuous PAP (CPAP) with a pressure of 4 cmH_2_O. CPAP was increased in 1 cm H_2_O increments in response to 2 apneas, 3 min of snoring, 3 hypopneas or 5 respiratory effort-related arousals (RERAs). If patients were unable to tolerate CPAP or if events still presented with CPAP at 15 cm H_2_O, the mode was switched to bilevel PAP. PAP titration was performed by a trained technologist. Sleep was staged according to the American Academy of Sleep Medicine (AASM) Manual for the Scoring of Sleep and Associated Events^18^. Obstructive apneas were defined as cessation of airflow for at least 10 s. Hypopneas were defined as a 30% reduction in nasal airflow from baseline for at least 10 s, associated with desaturation of at least 3% and/or an arousal on the electroencephalogram. Respiratory effort-related arousal was defined as a sequence of breaths lasting ≥ 10 s that were characterized by increasing respiratory effort, a flattening of the inspiratory portion of the nasal pressure (diagnostic study), or a PAP device flow (titration study) waveform leading to arousal from sleep when the sequence of breaths did not meet criteria for apnea or hypopnea. All sleep studies were scored by trained sleep technologists.

### Statistical analysis

SPSS version 22.0 software (SPSS Inc., Chicago, IL, USA) was used for all statistical analyses. All figures were carried out using GraphPad Prism 7.0 software (GraphPad Prism, Ver 7.0). Microsoft Word (Microsoft Office 2016, Microsoft Corporation, Redmond, Washington, USA) was used to design the flow chart. GraphPad Prism 7.0 software (GraphPad Software, Inc., La Jolla, California, USA) was used to generate the graphs. The Shapiro–Wilk test was used to assess the normality of all data. Independent-sample t test or the Mann–Whitney U test was used to compare demographic, clinical diagnostic and pressure titration PSG parameters in SWS rebounders versus non-SWS rebounders, and REM rebounders versus non-REM rebounders for normally and non-normally distributed continuous variables, respectively. A paired t test or the Wilcoxon signed rank sum test was used to compare PSG variables in the diagnostic PSG and pressure titration studies for normally and non-normally distributed continuous variables, respectively. SWS rebound and REM rebound were defined according to the differences in the REM proportion (%REM) or N3 proportion (%N3) between the pressure titration and diagnostic PSG (change in %SWS = %N3 in pressure titration—%N3 in the diagnostic test; change in %REM = %REM in pressure titration—%REM in the diagnostic test) and the values of the %REM or %N3 in pressure titration PSG. K-means clustering analysis was used to cluster the change in REM sleep characterized by %REM in pressure titration and the change in %REM, and change in SWS characterized by %N3 in pressure titration and the change in %N3. The integers for both the minimum %REM in the pressure titration study and minimum positive change in %REM in the more REM group served as cutoff values for REM sleep with or without rebound. The integers for both the minimum %N3 in the pressure titration study and minimum positive change in %N3 in the more SWS rebound group served as cutoff values for SWS with or without rebound. The χ^2^ test or Fisher’s test was used to compare proportions. Variables that were statistically significant after comparison between REM rebounders and non-REM rebounders and between SWS rebounders and non–SWS rebounders were entered into multiple linear stepwise regression analyses to determine the best model for predicting changes in REM sleep and SWS. The model with the highest adjusted R^2^ value was accepted as the best model predicting changes in REM sleep and SWS. A p value < 0.05 was considered statistically significant.

### Ethical approval

All the procedures involving human participants were performed in accordance with the ethical standards of the institutional and/or national research committee and the 1964 Declaration of Helsinki and its later amendments or comparable ethical standards. The study was approved by the Independent Ethics Committee of the Institution for National Drug Clinical Trials, Tangdu Hospital, Fourth Military Medical University. As this study employed a retrospective design with no violation of patient privacy, the ethics committee waived the requirement for informed consent.

## Results

### Difference in clinical data and PSG

Of the 501 enrolled patients, the average age was 49.87 ± 11.09 years, the average BMI was 29.12 ± 4.08 kg/m^2^, and 441 were men (88.02%). Compared with the diagnostic PSG study, patients in the pressure titration PSG study showed significant reductions in the total sleep time (TST) (p < 0.001), SE (p < 0.001), arousal index (p < 0.001), AHI (p < 0.001), oxygen desaturation index (ODI) (p < 0.001), total duration of apnea and hypopnea events (p < 0.001), mean duration of apnea and hypopnea events (p < 0.001), % N1 (p < 0.001), %N2 (p = 0.016), overall increase in sleep latency (SL) (p < 0.001), WASO (p = 0.045), %SWS (p < 0.001), % REM (p < 0.001) and oxygen saturation (p < 0.001) (Table 1). The %N3 and %REM differed between the pressure titration study and diagnostic polysomnography (Supplement Figs. 1 and 2).

**Table 1:** Differences between diagnostic and pressure titration PSG.

	Diagnostic PSG n = 501	Pressure titration study n = 501	Mean difference n = 501	p value
	Median	Median	Median	
TST, minutes	430.00 (390.25,456.50)	409.00 (368.25,440.50)	− 19.50 (− 62.50,16.75)	0.000***
SL, minutes	10.50 (5.00,19.50)	12.00 (6.50,22.50)	1.50 (− 5.00,10.50)	0.000***
SE, %	87.76 (79.60,93.10)	85.76 (77.05,92.36)	− 1.12 (− 7.78,3.99)	0.000***
WASO, minutes	46.00 (23.50,82.18)	49.50 (24.50,87.75)	2.50 (− 22.25,32.27)	0.045*
Arousal Index	9.25 (2.48,28.25)	0.90 (0.30,2.30)	− 4.40 (− 22.45,0.00)	0.000***
N1 duration, minutes	116.00 (75.25,174.75)	66.50 (45.00,100.25)	− 42.50 (− 103.00,− 8.25)	0.000***
N2 duration, minutes	221.00 (158.50,262.75)	195.00 (148.75,230.75)	− 20.00 (− 67.25,35.75)	0.000***
N3 duration, minutes	1.00 (0.00,23.50)	37.00 (16.00,63.25)	22.50 (1.50,48.25)	0.000***
REM duration, minutes	58.50 (40.25,77.00)	81.50 (57.50,108.25)	21.00 (− 4.50,51.50)	0.000***
%N1	27.80 (18.67,42.31)	16.98 (11.17,25.54)	− 10.03 (− 22.81,− 0.88)	0.000***
%N2	52.98 (41.17,61.25)	49.20 (39.74,57.39)	− 1.99 (− 13.10,9.03)	0.016*
%N3	0.21 (0.00,5.72)	9.78 (4.18,15.80)	5.87 (0.58,12.01)	0.000***
%REM	13.72 (10.26,17.57)	20.08 (15.69,26.01)	5.48 (0.64,13.21)	0.000***
AHI	50.05 (34.34,68.20)	1.52 (0.26,4.19)	− 47.20 (− 63.48,− 30.87)	0.000***
NAHI	50.06 (33.74,69.81)	1.52 (0.19,4.64)	− 47.20 (− 64.91,− 30.50)	0.000***
RAHI	50.96 (32.14,64.39)	0.46 (0.00,2.41)	− 47.54 (− 62.86,− 29.02)	0.000***
Mean duration of AH, minutes	0.39 (0.32,0.49)	0.27 (0.20,0.33)	− 0.14 (− 0.28,− 0.05)	0.000***
Total duration of AH, minutes	132.00 (76.70,236.20)	2.80 (0.40,8.35)	− 128.30 (− 221.35,− 69.55)	0.000***
Mean SpO_2_	93.00 (90.00,94.00)	96.00 (95.00,97.00)	3.00 (2.00,5.00)	0.000***
Nadir SpO_2_	73.00 (60.00,81.00)	89.00 (85.00,92.00)	15.00 (9.00,26.00)	0.000***
ODI	38.30 (18.01,65.18)	1.18 (0.00,4.95)	− 34.50 (− 58.95,− 16.42)	0.000***

*AH* apnea and hypopnea, *AHI* apnea–hypopnea index, *NREM* non-rapid eye movement sleep, *NAHI* non-rapid eye movement sleep apnea–hypopnea index, *ODI* oxygen desaturation index, *RAHI* non-rapid eye movement sleep apnea–hypopnea index, *REMs* rapid eye movement sleep, *SE* Sleep efficiency, *SL* sleep latency, *SpO*_*2*_ pulse oxygen saturation, *TST* Total sleep time, *WASO* wake after sleep onset.

### REM rebound and prediction model

The mean %REM in the pressure titration study was 28.62% (min–max: 16.44–51.88%), and the mean change in %REM between the pressure titration study and diagnostic PSG was 16.42% (min–max: 5.74–41.88%) in one cluster with more REM sleep determined using the K-means clustering analysis. The mean %REM in the pressure titration study was 16.13% (min–max: 0–27.95%), and the mean change in %REM between the pressure titration study and diagnostic PSG was 1.20% (min–max: − 21.84–12.01%) in the other cluster with less REM sleep. Therefore, for clinical communication, considering the integer of the more REM sleep group, REM rebound was defined as at least 16% in %REM in pressure titration PSG and an increase of at least 6% in %REM (Supplement Fig. 1). For the 225 (44.91%) patients that displayed REM rebound, the mean %REM on diagnostic PSG was 12.07% (mean duration of REM: 51.80 min), increasing to 27.27% (mean duration of REM: 113.15 min) on the pressure titration night.

Compared with non-REM rebounders, REM rebounders experienced significantly less insomnia (p = 0.004), including early insomnia (p < 0.001) and late insomnia (p = 0.047), and less dizziness (p = 0.011), anxiety (p < 0.001), irritability (p = 0.008), and depression (p = 0.023) (Table 2).

**Table 2:** The differences of clinical data between REM rebounders and non-REM rebounders.

	REM rebounders n = 225		Non-REM rebounders n = 276		χ^2^	p value
Age	48.36 ± 10.63	48.00 (41.00,55.50)	51.24 ± 11.24	51.00 (43.00,59.00)		0.006*
Sex (Male)	200	(88.9%)	241	(87.3%)		0.290
BMI	30.25 ± 4.24	29.94 (27.27,32.93)	28.34 ± 3.63	28.35 (25.88,30.48)		0.000***
ESS	12.98 ± 6.93	13.00 (7.00,19.00)	9.00 ± 6.15	8.00 (4.00,13.00)		0.000***
Insomnia	87	(38.67%)	142	(51.45%)	8.161	0.004**
Early insomnia	32	(14.22%)	75	(27.17%)	12.379	0.000***
Middle insomnia	58	(25.78%)	82	(29.71%)	0.952	0.329
Late insomnia	68	(24.63%)	39	(17.33%)	3.937	0.047*
Dizzy	86	(38.22%)	137	(49.64%)	6.54	0.011*
Anxiety	42	(18.67%)	92	(33.33%)	13.609	0.000***
Irritability	58	(25.78%)	102	(36.96%)	7.126	0.008**
Depression	49	(21.78%)	85	(30.80%)	5.146	0.023*
Cognition dysfunction	26	(11.56%)	45	(16.30%)	2.298	0.13
Gastric reflux	38	(16.89%)	41	(14.86%)	0.386	0.534
Hyposexuality	67	(29.78%)	61	(22.10%)	3.84	0.05
Fatigue	79	(35.11%)	101	(36.59%)	0.118	0.731
Hypertension	110	(48.89%)	128	(46.38%)	0.314	0.575
Cardiac Diseases	24	(10.67%)	42	(15.22%)	2.244	0.134
Diabetes mellitus	17	(7.56%)	27	(9.78%)	0.767	0.381
Legs uncomfortable	55	(24.44%)	74	(26.81%)	0.363	0.547
Stroke	11	(4.89%)	17	(6.16%)	0.379	0.538

**Figure 2:**
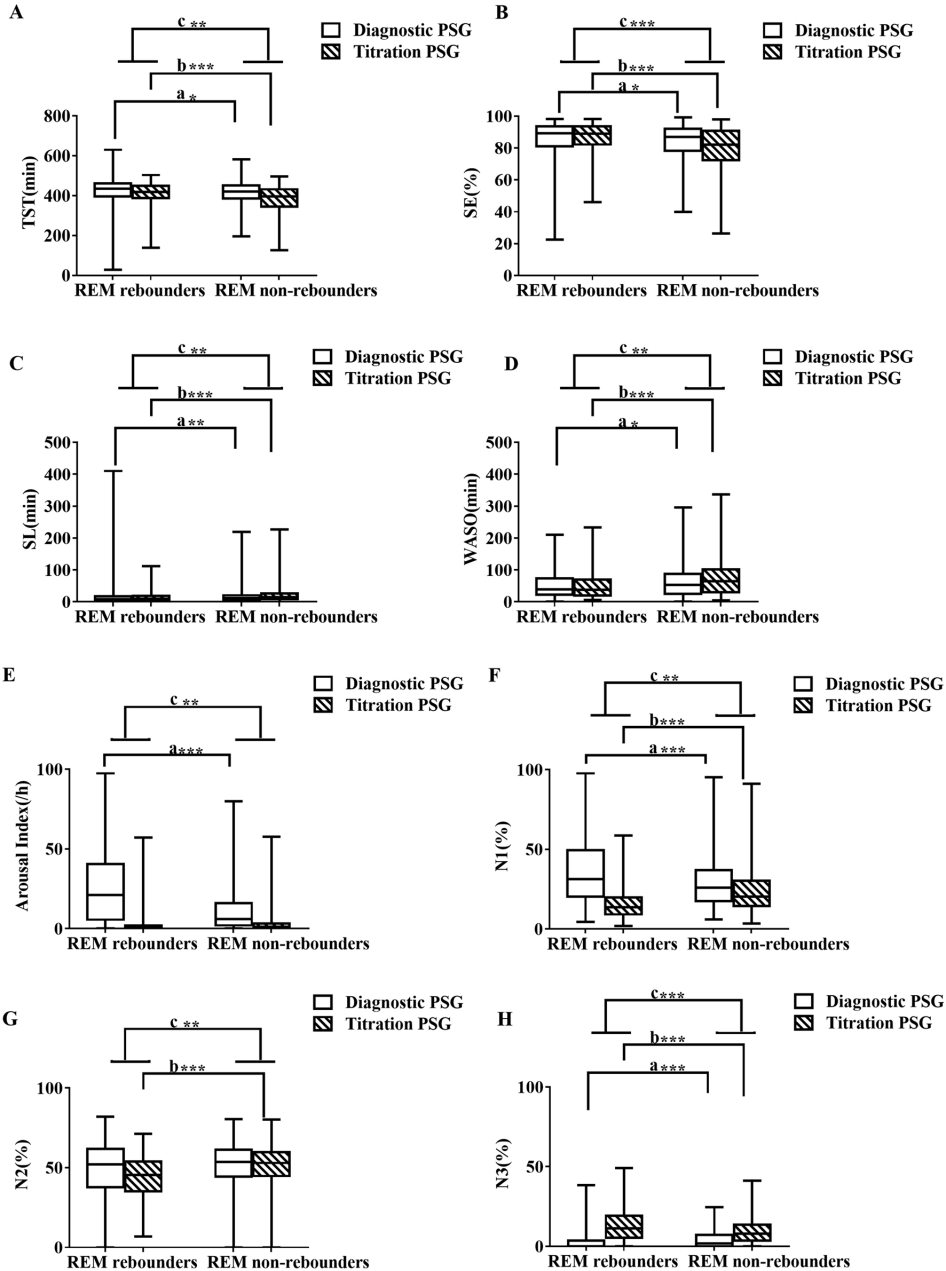

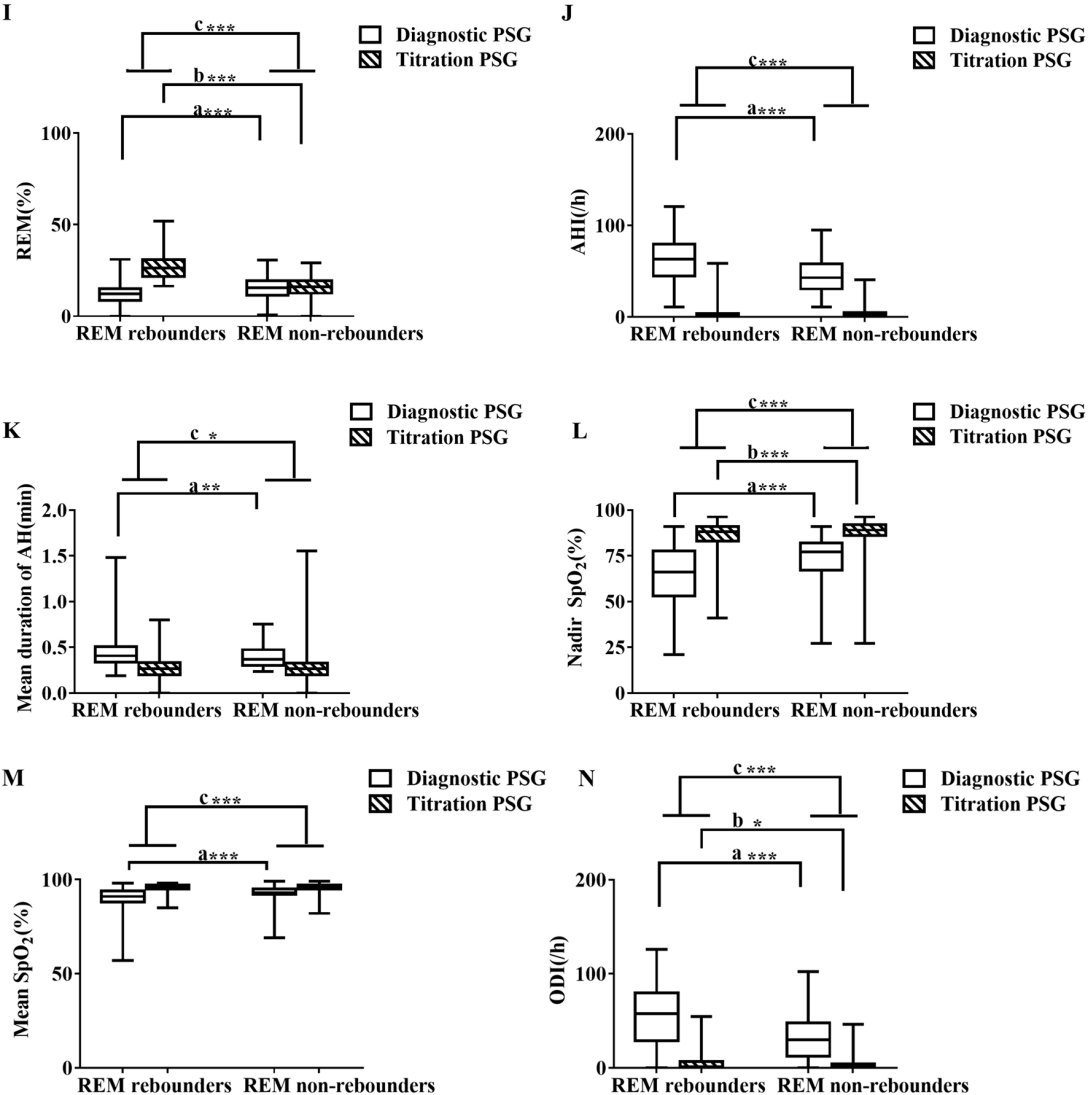
The changes from diagnostic PSG to pressure titration PSG between REM rebounders and non-REM rebounders. The figures showed the changes of diagnostic and pressure titration PSG in TST (A), SE (**B**), SL (**C**), WASO (**D**), Arousal Index (**E**), %N1 (**F**), %N2 (**G**), % N3 (**H**), % REM (**I**), AHI (**J**), Mean duration of apneas and hypopneas (**K**), Nadir SpO_2_ (**L**), Mean SpO_2_ (**M**), ODI (**N**) between REM rebounders and non-REM rebounders. a, comparison of diagnostic PSG parameters between REM rebounders and non-REM; b, comparison of pressure titration PSG parameters between REM rebounders and non–REM rebounders; c, comparison of the changes from pressure titration to diagnostic PSG parameters between REM rebounders and non-REM rebounders and * p < 0.05, **p < 0.01, ***p < 0.001. *AHI* apnea–hypopnea index, *BMI* body mass index, *ODI* oxygen desaturation index, *PSG* polysomnography, *REM* rapid eye movement sleep, *SE* sleep efficiency, *SL* sleep latency, *SWS* slow wave sleep, *SpO*_*2*_ pulse oxygen saturation, *TST* total sleep time, *WASO* wake after sleep onset, *REM* rapid eye movement sleep.

**Figure 3:**
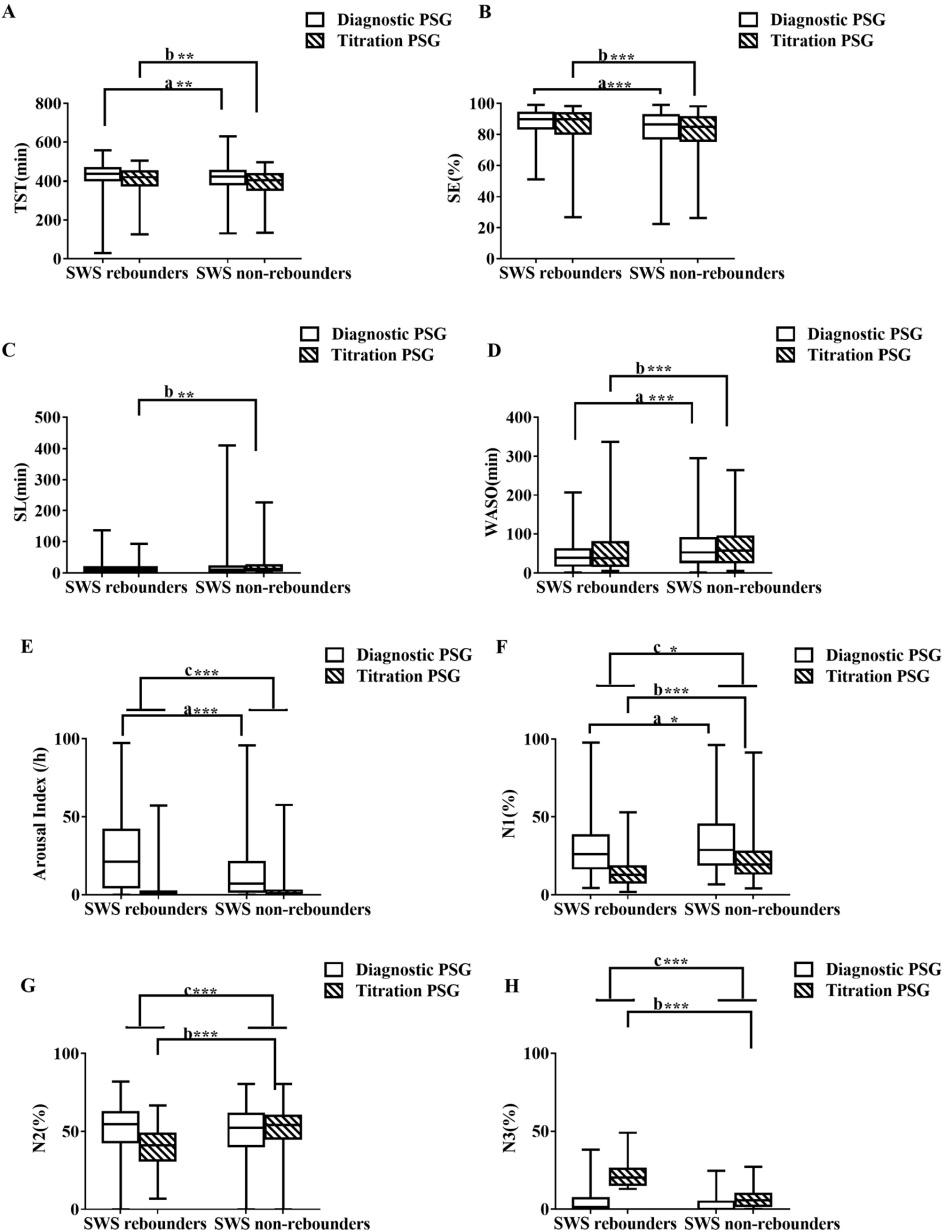

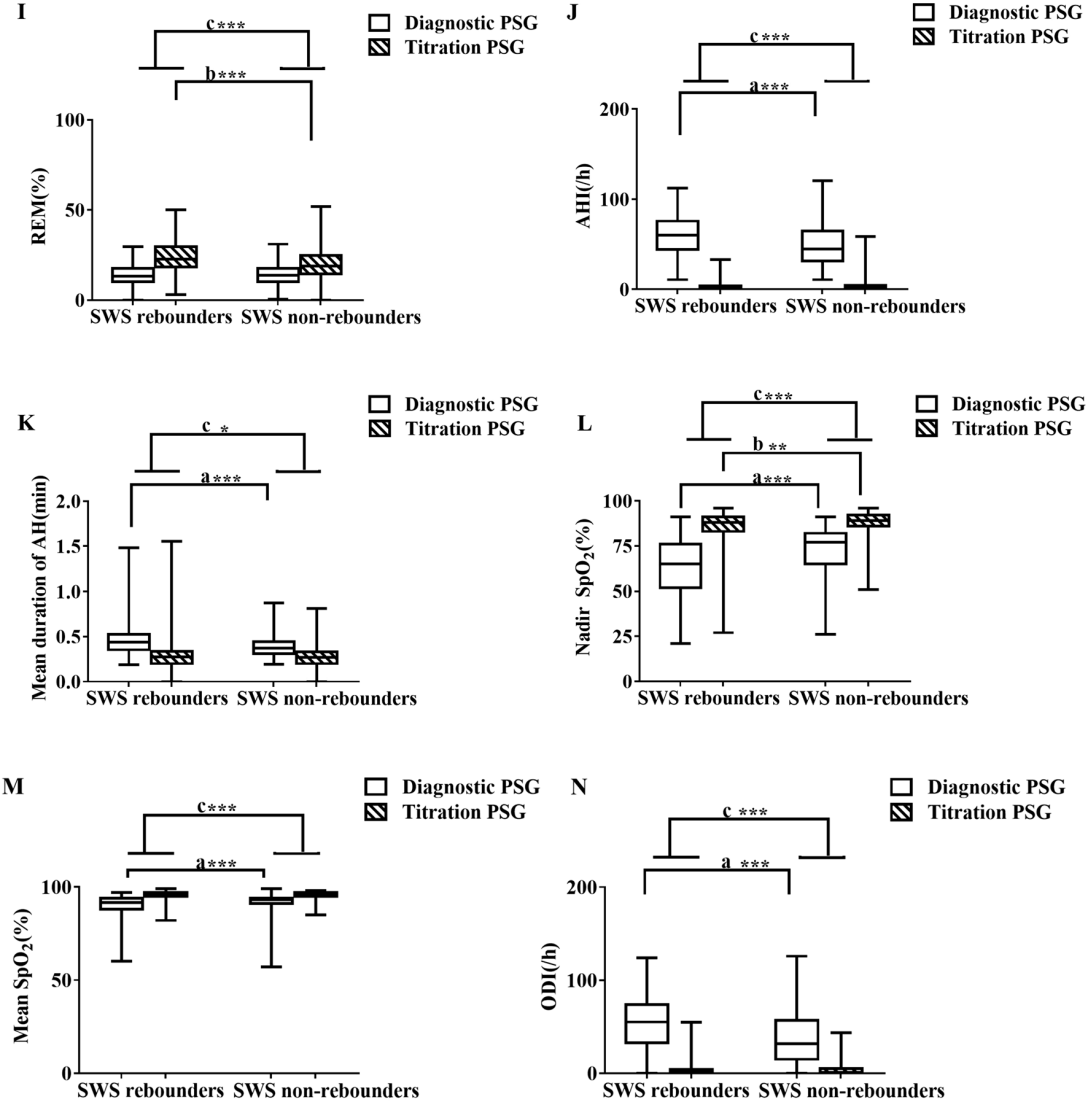
The change from diagnostic PSG to pressure titration PSG between and SWS rebounders and non-SWS rebounders. The figures showed the change of diagnostic and pressure titration PSG in TST (**A**), SE (**B**), SL (**C**), WASO (**D**), Arousal Index (**E**), %N1 (**F**), %N2 (**G**), % N3 (**H**), %REM (**I**), AHI (**J**), Mean duration of apneas and hypopneas (**K**), Nadir SpO_2_ (**L**), Mean SpO_2_ (**M**), ODI (**N**) between SWS rebounders and non–SWS rebounders. a, comparison of diagnostic PSG parameters between SWS rebounders and non-SWS rebounders; b, comparison of pressure titration PSG parameters between SWS rebounders and non-SWS rebounders; c, comparison of the change from pressure titration to diagnostic PSG parameters between SWS rebounders and non-SWS rebounders. * < 0.05, ** < 0.01, *** < 0.001. *AHI* apnea–hypopnea index, *BMI* body mass index, *ODI* oxygen desaturation index, *PSG* polysomnography, *REM* rapid eye movement sleep, *SE* sleep efficiency, *SL* sleep latency, *SWS* slow wave sleep, *SpO*_*2*_ pulse oxygen saturation, *TST* total sleep time, *WASO* wake after sleep onset.

Compared with non-REM rebounders, REM rebounders in the diagnostic PSG study had significantly more TST (p = 0.03), a shorter SL (p = 0.009), a higher SE (p = 0.012), less %N3 (p < 0.001) and %REM (p < 0.001), less WASO (p = 0.026), a higher arousal index (p < 0.001), a lower mean oxygen saturation (p < 0.001), a longer duration of apnea and hypopnea events (p < 0.001), a lower oxygen saturation nadir (p < 0.001), a higher AHI (p < 0.001) and ODI (p < 0.001) during the diagnostic PSG, a higher BMI (p < 0.001) and ESS (p < 0.001), and were younger (p = 0.006), without a difference in sex (p = 0.290) (Fig. 2 and Supplement Table 1). Compared with non-REM rebounders, REM rebounders in the pressure titration PSG study did not exhibit no differences in AHI (p = 0.384), but had more TST (p < 0.001), a shorter SL (p < 0.001), a higher SE (p < 0.001), less %N1 (p < 0.001), more %N2 (p < 0.009), %N3 (p < 0.001) and %REM (p < 0.001), less WASO (p < 0.001), more ODI (p = 0.027), and a lower oxygen saturation nadir (p < 0.001) (Fig. 3 and Supplement Table 2).

Compared with non-REM rebounders, REM rebounders had a significantly lower decrease in TST (p = 0.001), greater increase in SE (p < 0.001),lower increase in SL (p = 0.02), larger decrease in %N1 (p < 0.001) and %N2 (p = 0.001), larger increase in %N3 (p < 0.001) and %REM (p < 0.001), greater decrease in WASO (p = 0.001), greater decrease in arousal index (p < 0.001), greater decrease in AHI (p < 0.001), larger decrease in the ODI (p < 0.001), greater increase in oxygen saturation (p < 0.001), and greater increase in theSpO_2_ nadir (p < 0.001) in the change from diagnostic PSG to pressure titration PSG study (Fig. 3 and Supplement Table 2).

Predictors such as a younger age, higher ESS, more ODI, less %REM, lower mean oxygen saturation, and higher arousal index during diagnostic PSG together comprised the best model for predicting an increased change in %REM (R = 0.7, R^2^ = 0.489, adjusted R^2^ = 0.482, F = 69.950, p < 0.001) (Table 3).

**Table 3:** Model for the prediction of change in REM sleep.

Variables	Beta	95% CI	P value
Constant		(25.276,60.191)	< 0.001
ODI	0.21	(0.038,0.097)	< 0.001
%REM	− 0.442	(− 0.843,− 0.615)	< 0.001
Arousal index	0.154	(0.03,0.111)	0.001
MeanSpO_2_	− 0.141	(− 0.475,− 0.121)	0.001
ESS	0.115	(0.056,0.27)	0.003
Age	− 0.103	(− 0.149,− 0.027)	0.005

*CI* confidence interval, *ESS* Epworth sleepiness score, *ODI* oxygen desaturation index, *SpO*_*2*_ pulse oxygen saturation, *REM%* percentages of rapid eye movement sleep.

### SWS rebound and prediction model

After the K-means clustering analysis, the mean %N3 in the pressure titration study was 6.81% (min–max: 0–21.44%), and the mean change in %N3 was 3.4% (min–max: − 12.77%, 12.91%) in one cluster with less N3. The mean of %N3 in the pressure titration study was 23.06% (min–max: 13.39–49.04%), and the mean of the change in %N3 was 19.30% (min–max: − 4.1%, 2.63–49.04%) in the other cluster of more N3. The patient with a minimum positive increase in %N3 was 2.63%. Therefore, considering the integer in the more SWS group, N3 rebound was defined as at least 13% of %N3 in the pressure titration study and an increase of 3% in %N3 (Supplement Fig. 4). One hundred sixty-three (32.73%) patients exhibited SWS rebound. Ninety-four (18.76%) patients presented both REM rebound and SWS rebound, 131 showed (26.15%) only REM rebound, 70 (13.97%) exhibited only SWS rebound, and 206 (41.12%) experienced neither REM rebound nor SWS rebound. The mean %N3 in the diagnostic PSG study was 4.18% (mean duration of N3: 17.56 min) compared with 21.55% (mean duration of N3: 86.79 min) in the pressure titration study.

Compared with non-SWS rebounders, SWS rebounders had significantly less insomnia (p = 0.001), including early insomnia (p = 0.01) and late insomnia (p < 0.001), and less dizziness (p = 0.001), anxiety (p = 0.011), and irritability (p = 0.034) (Table 4). Compared with non-SWS rebounders, SWS rebounders had significantly more TST (p = 0.002), a higher SE (p < 0.001), less %N1 (p = 0.027), less WASO (p < 0.001), a higher arousal index (p < 0.001), a higher AHI (p < 0.001) and ODI (p < 0.001), a lower mean oxygen saturation (p < 0.001), a more severe reduction in the oxygen saturation nadir (p < 0.001) in the diagnostic PSG study, a higher BMI (p < 0.001) and ESS (p = 0.001), and were younger (p < 0.001) (Fig. 3 and Supplement Table 4). Compared with non-SWS rebounders, SWS rebounders showed no difference in AHI (p = 0.756), but significantly more TST (p = 0.001), less SL (p = 0.001), higher SE (p < 0.001), less N1 (p < 0.001) and N2 (p < 0.001), more N3 (p < 0.001) and REM sleep (p < 0.001), less WASO (p < 0.001), a lower mean oxygen saturation (p = 0.893), a lower oxygen saturation nadir (p = 0.001), and less ODI (p = 0.758) in the pressure titration PSG study (Fig. 3 and Supplement Table 5). Compared to non-SWS rebounders, SWS rebounders presented a significantly larger decrease in %N1 (p = 0.023) and %N2 (p < 0.001), greater increase in %N3 (p < 0.001) and %REM (p < 0.001), greater decrease in arousal index (p < 0.001), AHI (p < 0.001), ODI (p < 0.001) and mean duration of apnea and hypopnea events (p = 0.029), greater increase in SpO_2_ nadirs (p < 0.001) and mean SpO_2_ (p < 0.001), with no difference in TST, SL, SE and WASO in the changing from diagnostic PSG to pressure titration PSG (Fig. 3 and Supplement Table 6).

**Table 4:** Showed the differences of clinical data between SWS rebounders and non-SWS rebounders.

	SWS rebounders n = 164	Non-SWS rebounders n = 337	χ^2^	p value
Age	46.48 ± 10.49, 46.00 (39.00, 53.00)	51.58 ± 10.94, 51.00 (43.00, 59.00)		0.000***
Sex (Male)	146 (89.6%)	295 (87.5%)		0.509
BMI	30.34 ± 4.20 30.10 (27.77, 32.65)	28.64 ± 3.83 28.28 (26.08, 31.13)		0.000***
ESS	12.30 ± 6.80 11.00 (7.00, 18.00)	10.03 ± 6.75 9.00 (5.00, 14.75)		0.000***
Insomnia	57 (34.76%)	172 (51.04%)	11.786	0.001**
Early insomnia	24 (14.63%)	83 (24.63%)	6.561	0.01*
Middle insomnia	44 (26.83%)	96 (28.49%)	0.15	0.698
Late insomnia	20 (12.20%)	87 (25.82%)	12.185	0.000***
Dizzy	56 (34.14%)	167 (49.55%)	10.604	0.001**
Anxiety	32 (19.51%)	102 (30.27%)	6.513	0.011*
Irritability	42 (25.61%)	118 (35.01%)	4.489	0.034*
Depression	42 (25.61%)	92 (27.30%)	0.161	0.688
Cognition dysfunction	23 (14.02%)	48 (14.24%)	0.004	0.947
Gastric reflux	27 (16.46%)	52 (15.43%)	0.766	0.766
Hyposexuality	45 (27.44%)	83 (24.63%)	0.458	0.499
Fatigue	62 (37.80%)	118 (35.01%)	0.373	0.541
Hypertension	83 (50.61%)	155 (45.99%)	0.942	0.332
Cardiac diseases	23 (14.02%)	43 (12.76%)	0.154	0.694
Diabetes mellitus	15 (9.15%)	29 (8.61%)	0.04	0.841
Legs uncomfortable	43 (26.22%)	86 (25.52%)	0.028	0.866
Stroke	10 (6.10%)	18 (5.34%)	0.12	0.729

Together, a younger age, a longer total duration of apneas and hypopneas, shorter N3 duration, a lower SpO_2_ nadir, and less %REM sleep during diagnostic PSG produced the best model for predicting increases in %N3 between diagnostic and pressure titration PSG (R = 0.543, R^2^ = 0.295, adjusted R^2^ = 0.286, F = 36.657, p < 0.001) (Table 5).

**Table 5:** Model for the prediction of change in slow wave sleep.

Variables	Beta	95%, CI	P value
Constant		(25.276,60.191)	< 0.001
Total duration of apnea and hypopnea	0.320	(− 0.843,− 0.615)	< 0.001
N3 duration	− 0.214	(0.03,0.111)	< 0.001
Age	− 0.138	(− 0.475,− 0.121)	0.001
Nadir SpO_2_	− 0.122	(0.056,0.27)	0.020
%REM	− 0.082	(− 0.149,− 0.027)	0.048

*CI* confidence interval, *SpO*_*2*_ pulse oxygen saturation, *REM%* percentages of rapid eye movement sleep.

## Discussion

The cutoff values for REM sleep and SWS rebound in patients with OSA treated with PAP were identified using the objective statistical method K-means clustering analysis. In this study, REM sleep rebound was defined by at least 16% REM sleep on the pressure titration night and a 6% increase in %REM, whereas SWS rebound was defined by at least 13% N3 on the pressure titration night and a 3% increase of change in %N3. Two hundred twenty-five (44.91%) patients experienced REM rebound and 164 (32.73%) experienced SWS rebound. In a study by Osuna et al.^11^, 82/179 (46%) patients experienced REM rebound, similar to our study (44.91%). Osuna et al.^11^ also reported a 6% increase in %REM as a different point of REM rebound, which may have resulted in the similar prevalence of REM rebounders in our study; however, the authors did not indicate which statistical method was applied to obtain the cutoff value for the 6% change in %REM. In addition, Osuna et al.^11^ did not report a difference in the change in SWS between pressure titration and diagnostic PSG studies. In a study by Brillante et al.^8^, 76 of 335 patients (22.68%) experienced REM rebound, and 80 (23.8%) experienced SWS rebound, which was less than the values obtained in our study. The possible explanation for the difference may be that the definitions of REM rebound and SWS differed. Brillante et al.^8^ defined REM and SWS rebound according to the differences in REM sleep or SWS duration (in minutes) between pressure titration and diagnostic PSG studies as a percentage of the diagnostic PSG duration of REM sleep or SWS: (duration of REM sleep or SWS in diagnostic PSG—duration of REM sleep or SWS in pressure titration PSG)/duration of REM sleep or SWS in diagnostic PSG*100%. For patients who did not experience REM sleep or SWS during diagnostic PSG, a REM sleep or SWS period of > 15 min on the pressure titration night was considered a > 10% rebound. In patients diagnosed with OSA, SWS rebound was defined by a 40% increase on the pressure titration night compared with the diagnostic PSG night, whereas REM sleep rebound was defined by only a 20% increase. These thresholds were identified objectively using logarithmic equations and a forward sequential regression analysis^8^. Koo et al.^10^ found that 35/95 (36.84%) patients experienced REM rebound, and 17/95 (17.89%) experienced SWS rebound in the split night study. More REM sleep was supposed to occur during the last part of sleep when the pressure titration was performed in the split night study. Koo et al.^10^ reported a larger change in %REM between diagnostic and pressure titration study than was observed in our study (20% vs. 6%), which may contribute to a lower REM rebound prevalence than in our study. Koo et al.^10^ reported a lower change in SWS percentages between diagnostic and pressure titration studies than in our study (10% vs. 13%). The lower SWS rebound prevalence may be due to the lower amount of SWS during the last part of sleep when the pressure titration was performed in the split night study. Koo et al.^10^ used a split night study in OSA patients to define REM rebound using 2 criteria, including at least one REM period of ≥ 30 min duration and a ≥ 20% increase in REM sleep during the treatment portion. SWS rebound was defined as a ≥ 10% increase in SWS during the treatment portion; however, the study did not define the REM and SWS rebound threshold. The parameters age, %REM in diagnostic PSG, and the ODI, which is linearly related to AHI, were predictors of changes in REM and SWS in the current study, and BMI and AHI were considered predictors of changes in REM sleep in other studies^9^. Differences in the definitions of REM and SWS rebound, differences in the characteristics of patients with OSA (age: 47.0–58.6 years, BMI: 25.7–39.2 kg/m^2^, AHI 23.6–72.9 times/h) and %REM in diagnostic PSG (6.7–18.4%)^9^ lead to different prevalence rates of REM rebound and SWS rebound on the first night of exposure to PAP therapy.

No difference was observed in the AHI between the REM non-rebound and REM rebound groups during PAP treatment, suggesting that PAP treatment decreases respiratory events; however, differences were observed in the sleep architecture and clinical characteristics between the groups. These phenomena were similar between the SWS non-rebound and SWS rebound groups. According to the definition of REM rebound, compared with patients without REM rebound, patients with REM rebound were younger, had a higher BMI, severe ESS, more TST and SE, a short SL, a higher arousal index, less WASO, more N1 sleep, less REM sleep and SWS, and worse OSA (higher AHI and ODI, longer duration of apneas and hypopneas, and a lower baseline SpO_2_, SpO_2_ nadir, and degree of oxygen desaturation) in the diagnostic PSG study. These statistically significant differences were observed in most diagnostic PSG parameters that coincided with clinical symptoms. Compared with non-REM rebounders, REM rebounders experienced less dizziness, insomnia, anxiety, irritability, and depression. Similar to the present study, Koo et al.^10^ also found that patients with REM rebound tended to be younger, with a higher AHI and less REM sleep in the diagnostic portion than patients without REM rebound. In contrast to the present study, Koo et al.^10^ did not report t differences between the REM rebound and non-rebound groups in the degree of obesity, level of subjective sleepiness, or %SWS. Compared with patients without SWS rebound, patients with SWS rebound had more TST, higher SE, greater N2 and N3 sleep duration time, less N1%, less WASO, a higher arousal index, worse OSA (higher AHI, longer durations of apneas and hypopneas, lower mean oxygen saturation, substantial reduction in oxygen saturation during sleep, and a lower oxygen saturation nadir) in the diagnostic PSG study, a higher BMI and ESS, and were younger. Compared with patients without SWS rebound, patients with SWS rebound experienced less dizziness, insomnia, anxiety, and irritability. The cutoff values of 16% REM sleep in titration PSG and 6% changes in %REM were reasonable to define REM rebound and non-rebound according differences between the clinical and PSG characteristics. The SWS rebound cutoff value was also reasonable. Insomnia, anxiety, and depression can all lead to changes in sleep architecture^16,17^ and are common OSA comorbidities^12–15^. Thus, changes in the sleep architecture of patients with OSA are not caused by apnea and hypopnea events alone but also by comorbidities. The absence of REM and SWS rebound during the initial treatment of OSA with PAP may be explained by comorbid insomnia, anxiety, and depression. The initial PAP treatment can only decrease respiratory event-related arousal to restore REM sleep and SWS but has no effect on a longer sleep latency, increased WASO, decreased SWS, and REM sleep instability caused by insomnia, anxiety, or depression. Depression can increase REM sleep, explaining the greater incidence of depression in the REM non-rebound group, because patients with depression do not have a greater regulatory range of REM sleep^5^. Moreover, these patients may have increased sleep anxiety due to the use of PAP, resulting in a longer sleep latency, more WASO, and less SWS and REM sleep. The phenomena that patients with OSA but without REM rebound or SWS rebound reported more insomnia, anxiety and depression indicated that treatment of these comorbidities may possibly help restore the sleep architecture of patients with OSA through an initial pressure titration. This study is the first to explore the relationship among OSA comorbidities, REM and SWS rebound during initial pressure titration.

According to the clinical data and diagnostic PSG parameters, the present study established a predictive model of REM rebound and SWS rebound for patients with OSA undergoing PAP therapy. The ODI, %REM arousal index, baseline SpO_2_, ESS, and age were entered into the best model for predicting changes in REM sleep (adjusted R^2^ = 0.482), and the total duration of apneas and hypopneas, N3 duration, SpO_2_ nadir, REM% and age were entered into the best model for predicting changes in SWS (adjusted R^2^ = 0.286). According to the adjusted R^2^ values, the present prediction models explain a greater proportion of REM sleep variability than changes in SWS during titration PSG. Brillante et al.^8^ set REM rebound predictors with the AHI, %REM and arousal index (Cox and Snell R^2^ = 0.29) and SWS rebound predictors with age, BMI, SpO_2_ nadir, %N2, %SWS, and % REM (Cox and Snell R^2^ = 0.44). Osuna et al.^11^ found that REM rebound was predicted by variables such as REM sleep and the AHI at baseline PSG, as well as BMI, but did not observe SWS rebound in their study. Similar to the present study, AHI was not included among the predictors, but ODI was included (Pearson’s correlation coefficient between ODI and AHI 0.804, p < 0.001). The Instead of the ODI, the AHI with the variables %REM arousal index, baseline SpO_2_, ESS and age were analyzed using a multiple linear enter regression model, (R = 0.689, R^2^ = 0.475, adjusted R^2^ = 0.468). A higher AHI with %REM, lower arousal index, lower baseline SpO_2_, higher ESS and younger age can also be used to predict REM rebound, but the inclusion of the ODI in the prediction model produced better results than the model including the AHI in the prediction model (ODI adjusted R^2^ = 0.482, AHI adjusted R^2^ = 0.468). Generally, breath event-related parameters, such as a higher AHI, higher ODI and lower baseline SpO_2_, and sleep architecture parameters, such as a higher arousal index, lower %REM and younger age, are common variables for predicting REM rebound. Breath event-related parameters, such as a longer total duration of apnea and hypopnea events and lower SpO_2_ nadir, sleep architecture parameters such as a higher arousal index, less REM sleep and N3 duration, and younger, are common variables predicting SWS rebound.

### Limitations

The enrolled patients with OSA were derived from a single sleep medicine center in the Department of Neurology, indicating that most patients with OSA had neurological diseases and psychiatric diseases that could cause bias. Further studies are needed to verify whether the cutoff values for the definitions of REM and SWS rebound are suitable for all patients with OSA treated at the other sleep medicine centers. This study did not evaluate changes after-PAP treatment and did not analyze the clinical significance of REM or SWS rebound. All patients underwent only one diagnostic PSG and one pressure titration PSG; therefore, the first night effect of diagnostic and pressure titration were present, which may complicate the results.

## Conclusions

The combination of at least a 6% increase in %REM and 16% REM sleep during the pressure titration study reflected a significant REM rebound. The combination of at least a 3% increase in %SWS and 13% SWS during the pressure titration study reflected a significant SWS rebound. REM rebound was predicted by a higher ODI, lower %REM, higher arousal index and lower baseline SpO_2_ in the diagnostic PSG, higher ESS, and younger age (adjusted R^2^ = 0.482). SWS rebound was predicted by a younger age, longer total duration of apneas and hypopneas, shorter N3 duration, lower SpO_2_ nadir, and lower %REM in the diagnostic PSG (adjusted R^2^ = 0.286). Compared with SWS rebound, REM rebound was more obvious and prevalent during the pressure titration study. The diagnostic PSG and clinical data predict REM rebound better than SWS rebound. Compared with patients with REM rebound or SWS rebound, patients without REM rebound or SWS rebound had a higher probability of comorbidities with insomnia and mood complaints. The ability to predict which patients will experience REM and SWS rebound during pressure titration may help identify those who will be more likely to respond well to PAP therapy. The treatment of insomnia, anxiety and depression may restore the sleep architecture and improve compliance in patients with OSA who are treated with PAP.

## Supplementary Information

Supplementary Information.
