# Supplementary material for: Rapid eye movement sleep and slow wave sleep rebounded and related factors during positive airway pressure therapy
Source: Sci Rep. 2021 Apr 7;11:7599. doi: 10.1038/s41598-021-87149-3 (PMC8027864; doi:10.1038/s41598-021-87149-3)
Supplement: Supplementary file 1 — Supplementary Information. [file 41598_2021_87149_MOESM1_ESM.docx]

Rapid Eye Movement Sleep and Slow Wave Sleep rebounded and related factors during Positive Airway Pressure Therapy

Jin-xiang Cheng PhD MD^1^*, Jiafeng Ren MD^1^, Jian Qiu MD^1^, Yingcong Jiang MD^1^, Xianchao Zhao MD, Shuyu Sun MD^1^, Changjun Su MD^1^*

^1^Department of Neurology, Tangdu Hospital, Fourth Military Medical University, Xi’an 710038, Shaanxi Province, China

*Corresponding authors contributed equally

Changjun Su changjunsu@163.com, Department of Neurology, Tangdu Hospital, Fourth Military Medical University, Xi’an 710038, Shaanxi Province, China

Jin-xiang Cheng, chengjinxiang423@foxmail.com, Department of Neurology, Tangdu Hospital, Fourth Military Medical University, Xi’an 710038, Shaanxi Province, China

Supplement table 1 The differences of diagnostic PSG between REM rebounders and non–REM rebounders

|  | REM rebounders n=276 | Non-REM rebounders n=225 | p value | |
| --- | --- | --- | --- | --- |
|  | Median (25%, 75%) | Median (25%, 75%) |  |  |
| Age | 48.00(41.00,55.50) | 51.00(43.00,59.00) | 0.006 | * |
| Sex | 200（88.9%） | 241（87.3%） | 0.290 |  |
| BMI | 29.94(27.27,32.93) | 28.35(25.88,30.48) | 0.000 | *** |
| ESS | 13.00(7.00,19.00) | 8.00(4.00,13.00) | 0.000 | *** |
| TST, minutes | 435.00(396.00,461.25) | 421.25(386.00,452.50) | 0.030 | * |
| SL, minutes | 8.50(4.50,17.75) | 11.75(6.00,20.50) | 0.009 | ** |
| SE, % | 89.15(80.94,93.47) | 86.99(78.15,92.07) | 0.012 | * |
| WASO | 39.50(22.75,73.75) | 52.50(25.16,88.00) | 0.026 | * |
| Arousal Index | 21.10(5.65,40.45) | 5.95(2.00,16.00) | 0.000 | *** |
| Wake Index | 1.53(0.72,2.65) | 1.44(0.80,2.58) | 0.769 |  |
| N1 duration, minutes | 130.50(81.75,205.25) | 103.00(71.63,152.25) | 0.000 | *** |
| N2 duration, minutes | 214.50(142.75,266.50) | 222.25(164.63,258.25) | 0.622 |  |
| N3 duration, minutes | 0.00(0.00,14.50) | 7.75(0.00,31.00) | 0.000 | *** |
| REM duration, minutes | 53.50(34.50,67.50) | 65.00(44.13,86.38) | 0.000 | *** |
| N1% | 31.37(20.31,49.58) | 25.85(17.43,36.91) | 0.000 | *** |
| N2% | 51.97(37.69,61.77) | 53.70(44.31,61.23) | 0.255 |  |
| N3% | 0.00(0.00,3.52) | 1.88(0.00,7.21) | 0.000 | *** |
| REM% | 12.23(8.56,15.12) | 15.61(11.44,19.38) | 0.000 | *** |
| AHI | 63.17(44.80,79.55) | 42.87(30.59,58.13) | 0.000 | *** |
| NAHI | 63.79(43.71,81.11) | 41.81(30.35,59.32) | 0.000 | *** |
| RAHI | 58.79(41.63,70.48) | 44.16(26.05,59.93) | 0.000 | *** |

Supplement table 1 continued The differences of diagnostic PSG between REM rebounders and non–REM rebounders.

| Mean Duration of AH, minutes | 0.41(0.34,0.51) | 0.37(0.30,0.47) | 0.003 | ** |
| --- | --- | --- | --- | --- |
| Total Duration of AH, minutes | 186.10(100.30,280.70) | 108.20(68.15,175.95) | 0.000 | *** |
| Mean SpO_2_ | 91.00(88.00,94.00) | 93.00(92.00,95.00) | 0.000 | *** |
| Nadir SpO_2_ | 66.00(53.00,77.50) | 77.00(67.00,82.00) | 0.000 | *** |
| ODI | 57.47(28.60,80.04) | 30.00(12.29,47.78) | 0.000 | *** |

AH, apnea and hypopnea; AHI, apnea-hypopnea index; BMI, body mass index; ESS, Epworth sleepiness score; NREM, non-rapid eye movement sleep; NAHI, non-rapid eye movement sleep apnea-hypopnea index; ODI, oxygen desaturation index; RAHI, non-rapid eye movement sleep apnea-hypopnea index; REMs, rapid eye movement sleep; SE, Sleep efﬁciency; SL, sleep latency; SpO_2_, pulse oxygen saturation;TST, Total sleep time; WASO, wake after sleep onset

Supplement table 2 The differences of pressure titration PSG between REM rebounders and non–REM rebounders.

|  | REM rebounders n=276 | Non-REM rebounders n=225 | | p value | |
| --- | --- | --- | --- | --- | --- |
|  | Median (25%, 75%) | | Median (25%, 75%) |  |  |
| TST, minutes | 419.00(388.25,449.50) | | 396.25(345.00,432.38) | 0.000 | *** |
| SL, minutes | 10.00(5.00,18.00) | | 14.50(8.00,26.50) | 0.000 | *** |
| SE, % | 88.96(82.23,93.57) | | 82.10(72.33,90.73) | 0.000 | *** |
| WASO | 37.50(20.37,70.29) | | 64.25(29.63,101.50) | 0.000 | *** |
| Arousal Index | 0.90(0.30,1.90) | | 1.00(0.30,3.10) | 0.157 |  |
| N1 duration, minutes | 54.00(38.25,77.25) | | 78.25(53.00,108.50) | 0.000 | *** |
| N2 duration, minutes | 184.00(141.50,221.75) | | 202.25(153.13,241.25) | 0.002 | ** |
| N3 duration, minutes | 47.00(21.50,78.75) | | 31.00(13.00,53.38) | 0.000 | *** |
| REM duration, minutes | 108.50(86.75,133.00) | | 62.75(44.50,81.50) | 0.000 | *** |
| N1% | 13.62(9.24,19.86) | | 20.21(14.38,30.38) | 0.000 | *** |
| N2% | 45.36(35.31,53.75) | | 52.93(44.80,59.80) | 0.000 | *** |
| N3% | 11.31(5.48,19.21) | | 8.01(3.56,13.58) | 0.000 | *** |
| REM% | 26.38(21.72,30.99) | | 16.07(12.59,19.28) | 0.000 | *** |
| AHI | 1.48(0.28,3.60) | | 1.58(0.18,4.88) | 0.384 |  |
| NAHI | 1.58(0.22,3.89) | | 1.43(0.17,5.14) | 0.974 |  |
| RAHI | 0.48(0.00,1.84) | | 0.00(0.00,3.24) | 0.318 |  |
| Mean Duration of AH, minutes | 0.27(0.20,0.33) | | 0.27(0.20,0.33) | 0.836 |  |
| Total Duration of AH, minutes | 2.80(0.60,7.30) | | 2.80(0.40,9.88) | 0.721 |  |
| Mean SpO_2_ | 96.00(95.00,97.00) | | 96.00(95.00,97.00) | 0.422 |  |
| Nadir SpO_2_ | 88.00(83.00,91.00) | | 89.00(86.00,92.00) | 0.000 | *** |
| ODI | 1.42(0.13,6.59) | | 0.87(0.00,4.30) | 0.027 | * |

AH, apnea and hypopnea; AHI, apnea-hypopnea index; NREM, non-rapid eye movement sleep; NAHI, non-rapid eye movement sleep apnea-hypopnea index; ODI, oxygen desaturation index; RAHI, non-rapid eye movement sleep apnea-hypopnea index; REMs, rapid eye movement sleep; SE, Sleep efﬁciency; SL, sleep latency; SpO_2_, pulse oxygen saturation; TST, Total sleep time; WASO, wake after sleep onset

Supplement table 3 The change from diagnostic PSG to pressure titration PSG between REM rebounders and non-REM rebounders.

|  | | REM rebounders n=225 | | REM non-rebounders n=276 | | | p value | | |  |
| --- | --- | --- | --- | --- | --- | --- | --- | --- | --- | --- |
|  | | Median (25%, 75%) | | Median (25%, 75%) | |  | | |  |  |
| TST, minutes | | -14.50(-45.50,27.25) | | -22.50(-81.13,5.88) | | 0.001 | | | ** |  |
| SL, minutes | | 0.50(-5.00,6.50) | | 2.50(-5.00,13.38) | | 0.020 | | | * |  |
| SE, % | | -0.02(-5.34,5.84) | | -2.52(-10.04,2.87) | | 0.000 | | | *** |  |
| WASO | | -5.40(-18.25,-1.10) | | -9.95(-21.95,-2.65) | | 0.003 | | | ** |  |
| Arousal Index | | -1.50(-26.50,20.50) | | 9.25(-19.38,39.39) | | 0.001 | | | ** |  |
| N1 duration, minutes | | -69.00(-144.00,-29.25) | | -23.50(-66.38,0.88) | | 0.000 | | | *** |  |
| N2 duration, minutes | | -26.50(-86.00,40.25) | | -14.25(-60.88,30.88) | | 0.170 | | |  |  |
| N3 duration, minutes | | 32.00(13.00,62.75) | | 15.50(0.00,34.75) | | 0.000 | | | *** |  |
| REM duration, minutes | | 54.50(36.25,77.25) | | 0.00(-18.50,14.00) | | 0.000 | | | *** |  |
| N1% | | -16.80(-34.85,-7.67) | | -4.67(-14.63,2.59) | | 0.000 | | | *** |  |
| N2% | | -6.43(-17.48,9.26) | | -0.05(-8.63,8.96) | | 0.001 | | | ** |  |
| N3% | | 8.10(3.24,15.72) | | 4.28(0.00,9.33) | | 0.000 | | | *** |  |
| REM% | | 13.89(10.03,17.99) | | 1.18(-2.03,3.91) | | 0.000 | | | *** |  |
| AHI | | -59.39(-76.83,-42.16) | | -38.01(-53.68,-26.27) | | 0.000 | | | *** |  |
| NAHI | | -59.06(-77.31,-41.39) | | -38.44(-54.85,-26.32) | | 0.000 | | | *** |  |
| RAHI | | -57.45(-68.92,-37.87) | | -40.65(-56.29,-23.08) | | 0.000 | | | *** |  |
| Mean Duration of AH, minutes | | -0.16(-0.29,-0.07) | | -0.11(-0.28,-0.05) | | 0.040 | | | * |  |
| Total Duration of AH, minutes | | -365.00(-552.00,-178.00) | | -168.50(-318.75,-66.50) | | 0.000 | | | *** |  |
| Mean SpO_2_ | 4.00(2.50,7.00) | | 2.50(1.00,4.00) | | 0.000 | | | *** | | |
| Nadir SpO_2_ | 21.00(12.00,30.00) | | 12.00(7.00,20.00) | | 0.000 | | | *** | | |
| ODI | -54.20(-74.54,-26.87) | | -26.49(-44.71,-9.23) | | 0.000 | | | *** | | |

AH, apnea and hypopnea; AHI, apnea-hypopnea index; BMI, body mass index; ESS, Epworth sleepiness score; NREM, non-rapid eye movement sleep; NAHI, non-rapid eye movement sleep apnea-hypopnea index; ODI, oxygen desaturation index; RAHI, non-rapid eye movement sleep apnea-hypopnea index; REMs, rapid eye movement sleep; SE, Sleep efﬁciency; SL, sleep latency; SpO_2_, pulse oxygen saturation; TST, Total sleep time; WASO, wake after sleep onset

Supplement table 4 The differences of diagnostic PSG between SWS rebounders and Non–SWS rebounders.

|  | SWS rebounders n=164 | non-SWS rebounders n=337 | | | p value | |
| --- | --- | --- | --- | --- | --- | --- |
|  | Median (25%, 75%) | | Median (25%, 75%) |  | |  |
| Age | 46.00(39.00,53.00) | | 51.00(43.00,59.00) | 0.000 | | *** |
| Sex | 146 (89.6%) | | 295(87.5%) | 0.509 | |  |
| BMI | 30.10(27.77,32.65) | | 28.28(26.08,31.13) | 0.000 | | *** |
| ESS | 11.00(7.00,18.00) | | 9.00(5.00,14.75) | 0.000 | | *** |
| TST, minutes | 436.00(403.50,465.50) | | 421.50(385.50,451.25) | 0.001 | | ** |
| SL, minutes | 10.50(4.00,18.50) | | 10.50(5.25,20.50) | 0.002 | | ** |
| SE, % | 89.83(83.94,94.01) | | 86.46(77.55,92.33) | 0.277 | |  |
| WASO | 38.00(19.00,60.00) | | 52.13(27.75,88.85) | 0.000 | | *** |
| Arousal Index | 22.00(5.13,41.63) | | 7.20(2.00,21.00) | 0.000 | | *** |
| N1 duration, minutes | 107.50(69.50,162.50) | | 117.50(77.00,178.75) | 0.000 | | *** |
| N2 duration, minutes | 233.50(176.50,280.00) | | 212.00(153.75,256.75) | 0.086 | |  |
| N3 duration, minutes | 6.00(0.00,30.00) | | 0.00(0.00,20.50) | 0.002 | | ** |
| REM duration, minutes | 58.50(41.00,77.00) | | 58.50(40.00,76.50) | 0.043 | | * |
| N1% | 26.03(17.15,38.34) | | 28.76(19.35,45.04) | 0.644 | |  |
| N2% | 54.68(43.33,62.43) | | 52.18(40.60,61.20) | 0.027 | | * |
| N3% | 1.30(0.00,6.97) | | 0.00(0.00,4.85) | 0.052 | |  |
| REM% | 13.29(10.27,17.57) | | 13.78(10.21,17.57) | 0.052 | |  |
| AHI | 60.39(44.29,75.88) | | 44.70(31.45,64.57) | 0.677 | |  |
| NAHI | 62.27(44.01,77.39) | | 44.11(30.35,65.86) | 0.000 | | *** |
| RAHI | 54.64(38.67,68.57) | | 50.18(28.43,63.23) | 0.000 | | *** |

Supplement table 4 continued The differences of diagnostic PSG between SWS rebounders and Non–SWS rebounders.

| Mean Duration of AH, minutes | 0.44(0.36,0.52) | 0.37(0.31,0.44) | 0.004 | ** |
| --- | --- | --- | --- | --- |
| Total Duration of AH, minutes | 193.10(114.20,289.30) | 110.20(68.20,194.75) | 0.000 | *** |
| Mean SpO_2_ | 92.00(88.00,94.00) | 93.00(91.00,94.00) | 0.000 | *** |
| Nadir SpO_2_ | 65.00(52.00,76.00) | 77.00(65.00,82.00) | 0.000 | *** |
| ODI | 55.10(33.70,73.70) | 31.70(15.00,56.85) | 0.000 | *** |

AH, apnea and hypopnea；AHI, apnea-hypopnea index; BMI, body mass index; ESS,Epworth sleepiness score; NREM, non-rapid eye movement sleep; NAHI, non-rapid eye movement sleep apnea-hypopnea index; ODI, oxygen desaturation index; RAHI, non-rapid eye movement sleep apnea-hypopnea index; REMs, rapid eye movement sleep; SE, Sleep efﬁciency; SL, sleep latency; SpO_2_,pulse oxygen saturation; TST, Total sleep time; WASO, wake after sleep onset

Supplement table 5 The differences of pressure titration PSG between SWS rebounders and Non–SWS rebounders.

|  | SWS rebounders n=164 | Non–SWS rebounders n=337 | | | p value | |
| --- | --- | --- | --- | --- | --- | --- |
|  | Median (25%, 75%) | | Median (25%, 75%) |  | |  |
| TST, minutes | 418.50(379.00,449.00) | | 403.50(356.00,434.75) | 0.001 | | ** |
| SL, minutes | 10.50(4.50,18.00) | | 13.00(7.50,24.75) | 0.001 | | ** |
| SE, % | 89.88(80.40,93.68) | | 84.90(75.84,91.16) | 0.000 | | *** |
| WASO | 36.50(18.22,78.00) | | 56.50(27.50,93.25) | 0.000 | | *** |
| Arousal Index | 0.80(0.30,2.05) | | 1.00(0.30,2.70) | 0.213 | |  |
| N1 duration, minutes | 49.50(33.50,75.50) | | 73.00(51.75,105.75) | 0.000 | | *** |
| N2 duration, minutes | 167.50(124.50,201.00) | | 210.50(166.00,245.50) | 0.000 | | *** |
| N3 duration, minutes | 78.50(63.00,105.50) | | 23.50(9.00,37.75) | 0.000 | | *** |
| REM duration, minutes | 93.50(71.00,125.50) | | 74.00(54.50,101.00) | 0.000 | | *** |
| N1% | 12.90(8.10,18.13) | | 19.36(13.67,27.73) | 0.000 | | *** |
| N2% | 41.08(31.32,48.52) | | 54.02(45.42,59.89) | 0.000 | | *** |
| N3% | 20.20(15.73,25.69) | | 5.92(2.21,9.87) | 0.000 | | *** |
| REMs% | 22.75(18.29,29.18) | | 18.94(14.44,24.68) | 0.000 | | *** |
| AHI | 1.58(0.25,3.87) | | 1.44(0.26,4.29) | 0.756 | |  |
| NAHI | 1.66(0.21,4.12) | | 1.43(0.18,4.71) | 0.533 | |  |
| RAHI | 0.54(0.00,2.25) | | 0.00(0.00,2.52) | 0.951 | |  |
| Mean Duration of AH, minutes | 0.27(0.20,0.34) | | 0.27(0.20,0.33) | 0.472 | |  |
| Total Duration of AH, minutes | 22.52(0.50,8.20) | | 17.54(0.40,8.45) | 0.500 | |  |
| MeanSpO_2_ | 96.00(95.00,97.00) | | 96.00(95.00,97.00) | 0.893 | |  |
| Nadir SpO_2_ | 88.00(83.00,91.00) | | 89.00(86.00,92.00) | 0.001 | | ** |
| ODI | 7.42(0.00,4.20) | | 6.61(0.00,5.24) | 0.758 | |  |

AH, apnea and hypopnea; AHI, apnea-hypopnea index; BMI, body mass index; ESS, Epworth sleepiness score; NREM, non-rapid eye movement sleep; NAHI, non-rapid eye movement sleep apnea-hypopnea index; ODI, oxygen desaturation index; RAHI, non-rapid eye movement sleep apnea-hypopnea index; REMs, rapid eye movement sleep; SE, Sleep efﬁciency; SL, sleep latency; SpO_2_, pulse oxygen saturation;TST, Total sleep time; WASO, wake after sleep onset

Supplement table 6 The change from diagnostic PSG to pressure titration PSG between SWS rebounders and Non–SWS rebounders.

|  | SWS rebounders n=164 | Non-SWS rebounders n=337 | | p value |
| --- | --- | --- | --- | --- |
|  | Median (25%, 75%) | Median (25%, 75%) |  |  |
| TST, minutes | -18.00(-59.00,20.00) | -20.00(-64.00,16.00) | 0.577 |  |
| SL, minutes | 0.50(-6.50,8.50) | 2.00(-4.50,12.00) | 0.293 |  |
| SE, % | -0.72(-6.76,4.31) | -1.35(-8.15,3.88) | 0.403 |  |
| WASO | -4.30(-15.30,-0.90) | -9.98(-24.80,-2.90) | 0.000 | *** |
| Arousal Index | 2.00(-21.00,30.50) | 2.50(-22.50,33.50) | 0.830 |  |
| N1 duration, minutes | -49.50(-106.50,-21.50) | -37.50(-97.25,-4.00) | 0.027 | * |
| N2 duration, minutes | -57.00(-123.00,1.00) | -3.50(-51.75,51.25) | 0.000 | *** |
| N3 duration, minutes | 61.00(42.00,92.50) | 10.50(0.00,25.00) | 0.000 | *** |
| REM duration, minutes | 33.50(6.00,74.50) | 16.50(-6.25,42.50) | 0.000 | *** |
| N1% | -11.80(-23.43,-3.90) | -8.46(-21.99,0.18) | 0.023 | * |
| N2% | -11.73(-22.85,-0.35) | 0.63(-8.00,12.53) | 0.000 | *** |
| N3% | 15.70(10.20,22.76) | 2.63(0.00,6.63) | 0.000 | *** |
| REMs% | 9.56(2.40,17.67) | 4.81(0.10,10.89) | 0.000 | *** |
| AHI | -54.22(-70.54,-41.91) | -40.99(-60.87,-27.41) | 0.000 | *** |
| NAHI | -55.52(-71.79,-41.07) | -41.24(-60.99,-26.58) | 0.000 | *** |
| RAHI | -50.67(-66.44,-36.99) | -45.94(-60.71,-24.31) | 0.002 | ** |
| Mean Duration of AH, minutes | -0.17(-0.30,-0.07) | -0.12(-0.26,-0.05) | 0.029 | * |
| Total Duration of AH, minutes | -361.00(-518.00,-199.00) | -184.00(-360.00,-88.00) | 0.000 | *** |
| MeanSpO_2_ | 4.00(2.00,7.00) | 3.00(2.00,4.00) | 0.000 | *** |
| Nadir SpO_2_ | 21.00(12.00,31.00) | 13.00(8.00,22.00) | 0.000 | *** |
| ODI | -51.28(-69.65,-30.11) | -27.50(-51.28,-12.10) | 0.000 | *** |

AH, apnea and hypopnea; AHI, apnea-hypopnea index; BMI, body mass index; ESS, Epworth sleepiness score; NREM, non-rapid eye movement sleep; NAHI, non-rapid eye movement sleep apnea-hypopnea index; ODI, oxygen desaturation index; RAHI, non-rapid eye movement sleep apnea-hypopnea index; REMs, rapid eye movement sleep; SE, Sleep efﬁciency; SL, sleep latency; SpO_2_, pulse oxygen saturation; TST, Total sleep time; WASO, wake after sleep onset

Supplement figure 1 The change of %REM between pressure titration and diagnostic PSG.


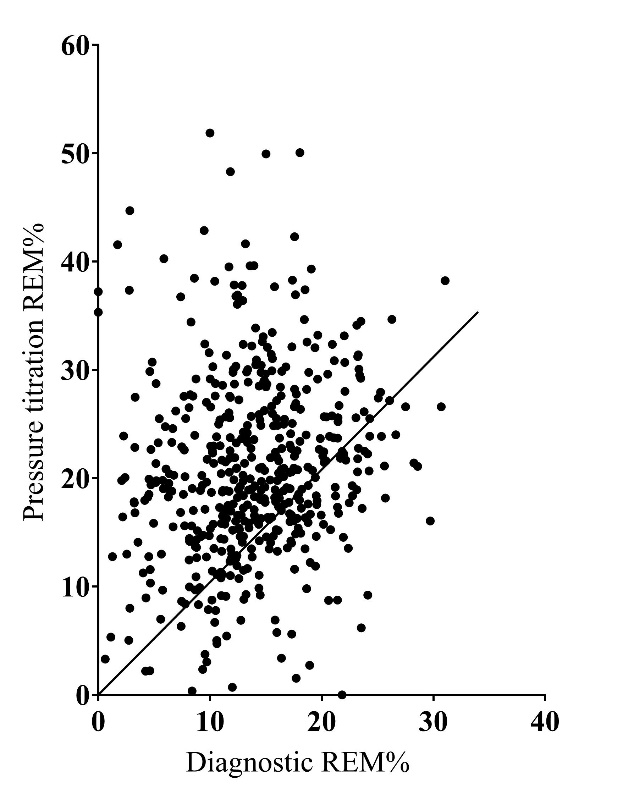


Different people showed different %REM change between diagnostic and pressure titration PSG. Every dot means a person, X axis is %REM in diagnostic PSG, Y axis is %REM in pressure titration PSG. Line is where %REM is the same between diagnostic and pressure titration PSG.

Supplement figure 2 The change of N3% between pressure titration and diagnostic PSG.


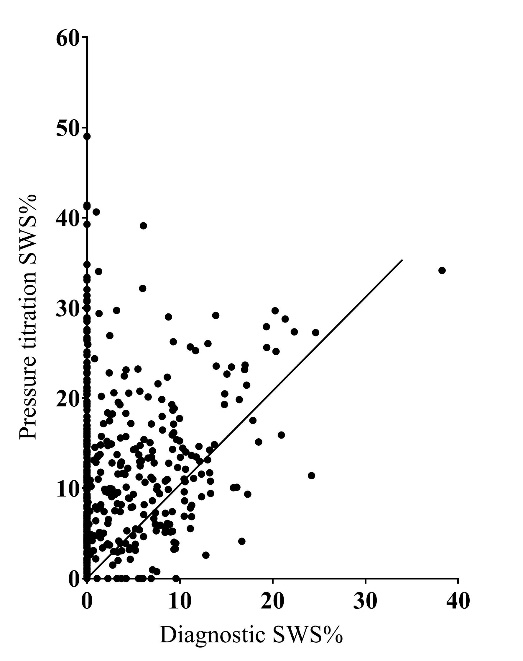


Different people showed different %N3 change between diagnostic and pressure titration PSG. Every dot means a person, X axis is %N3 in diagnostic PSG, Y axis is % N3in pressure titration PSG. Line is where %N3 is the same between diagnostic and pressure titration PSG.

Supplement figure 3 The clusters of K mean cluster analysis and the clusters of defined REM rebound and no- REM rebound.


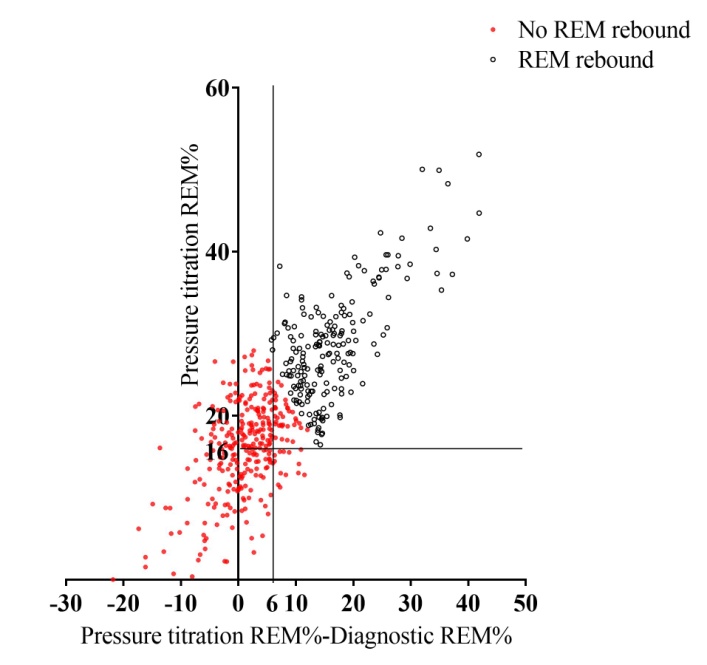


X axis is %REM in pressure titration PSG-%REM in diagnostic PSG, Y axis is %REM in pressure titration PSG. According to K-Mean cluster analysis, Red solid dots mean no REM rebound, black empty dots mean REM rebound. Cross + is line of the minimum of %REM in pressure titration PSG-%REM in diagnostic PSG 6%, and line of the minimum of %REM in pressure titration PSG 16% flowing K mean cluster. Clinical REM rebound and no REM rebound defined according to cutoff value 6% (%REM in pressure titration PSG-%REM in diagnostic PSG), and 16% REM sleep in pressure titration PSG. PSG, polysomnography. REM, rapid eye movement; %REM, rapid eye movement sleep duration percentage in total sleep time

Supplement figure 4 The clusters of K mean cluster and the clusters of defined SWS rebound and non-SWS rebound.


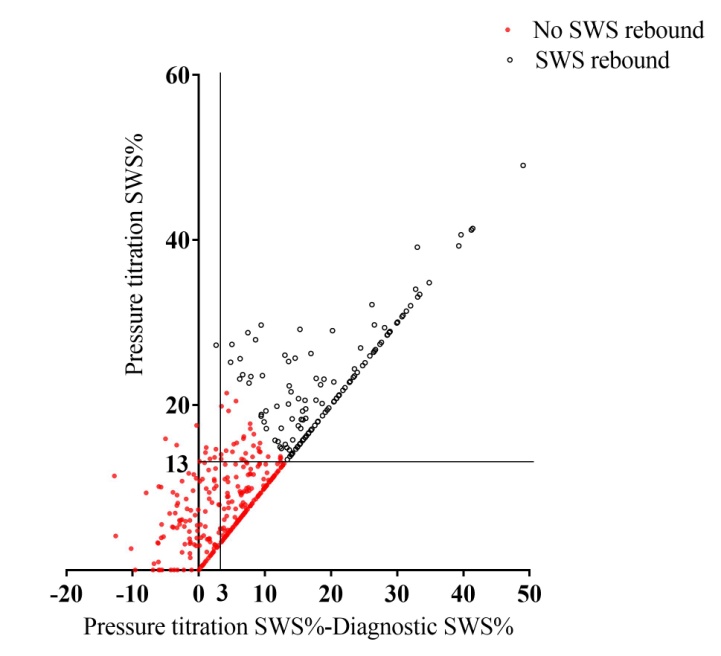


X axis is %%REM in pressure titration PSG-%%SWS in diagnostic PSG, Y axis is% %SWS in pressure titration PSG. According to K-Mean cluster analysis, Red solid dots mean no SWS rebound, black empty dots mean SWS rebound. Cross + is line of the minimum of %SWS in pressure titration PSG-%SWS in diagnostic PSG 6%, and line of the minimum of %SWS in pressure titration PSG 16% following K mean cluster. Clinical SWS rebound and no SWS rebound defined according to cutoff value 6% (%REM in pressure titration PSG-%REM in diagnostic PSG), and 16% REM sleep in pressure titration PSG. PSG, polysomnography; SWS, slow wave sleep; %SWS, slow wave sleep duration percentage in total sleep time
